# Supplementary material for: Variation in phenology of hibernation and reproduction in the endangered New Mexico meadow jumping mouse (Zapus hudsonius luteus)
Source: PeerJ. 2015 Aug 6;3:e1138. doi: 10.7717/peerj.1138 (PMC4540022; doi:10.7717/peerj.1138)
Supplement: Table S1 — Specimen data used in the evaluation of the phenology of hibernation and reproduction in the New Mexico meadow jumping mouse (Zapus hudsonius luteus). Museums acronyms are defined in text. Julian date is the date of capture. Age class is described in text. [file peerj-03-1138-s001.docx]

| **Group** | **Population** | **Museum** | **Specimen Number** | **State** | **County** | **Julian Date** | **Age Class** | **Sex** |
| --- | --- | --- | --- | --- | --- | --- | --- | --- |
| montane | Jemez Mountains | FT | 340 | NM | Sandoval | 180 | 0.44 | female |
| montane | Jemez Mountains | FT | 348 | NM | Sandoval | 180 | 0.50 | male |
| montane | Jemez Mountains | FT | 353 | NM | Sandoval | 182 | 0.44 | female |
| montane | Jemez Mountains | FT | 354 | NM | Sandoval | 182 | 0.50 | male |
| montane | Jemez Mountains | FT | 355 | NM | Sandoval | 185 | 0.50 | male |
| montane | Jemez Mountains | FT | 356 | NM | Sandoval | 186 | 0.35 | female |
| montane | Jemez Mountains | FT | 357 | NM | Sandoval | 186 | 0.35 | female |
| montane | Jemez Mountains | FT | 360 | NM | Sandoval | 179 | 0.71 | male |
| montane | Jemez Mountains | FT | 613 | NM | Sandoval | 227 | 0.44 | male |
| montane | Jemez Mountains | MSB | 41055 | NM | Sandoval | 217 | 0.35 | male |
| montane | Jemez Mountains | MSB | 56979 | NM | Sandoval | 239 | 0.35 | female |
| montane | Jemez Mountains | MSB | 56980 | NM | Sandoval | 240 | 0.35 | female |
| montane | Jemez Mountains | MSB | 56981 | NM | Sandoval | 240 | 0.47 | male |
| montane | Jemez Mountains | MSB | 56982 | NM | Sandoval | 235 | 0.50 | female |
| montane | Jemez Mountains | MSB | 56983 | NM | Sandoval | 239 | 0.35 | male |
| montane | Jemez Mountains | MSB | 56984 | NM | Sandoval | 239 | 0.15 | female |
| montane | Jemez Mountains | MSB | 56985 | NM | Sandoval | 242 | 0.35 | male |
| montane | Jemez Mountains | MSB | 56986 | NM | Sandoval | 249 |  | female |
| montane | Jemez Mountains | MSB | 56987 | NM | Sandoval | 249 | 0.21 | male |
| montane | Jemez Mountains | MSB | 56988 | NM | Sandoval | 249 | 0.15 | female |
| montane | Jemez Mountains | MSB | 56989 | NM | Sandoval | 249 | 0.15 | male |
| montane | Jemez Mountains | MSB | 56990 | NM | Sandoval | 248 | 0.58 | female |
| montane | Jemez Mountains | MSB | 56991 | NM | Sandoval | 248 | 0.35 | male |
| montane | Jemez Mountains | MSB | 56992 | NM | Sandoval | 248 | 0.35 | female |
| montane | Jemez Mountains | MSB | 56993 | NM | Sandoval | 235 | 0.35 | female |
| montane | Jemez Mountains | MSB | 56994 | NM | Sandoval | 239 | 0.42 | female |
| montane | Jemez Mountains | MSB | 62096 | NM | Sandoval | 214 | 0.35 | male |
| montane | Jemez Mountains | MSB | 62097 | NM | Sandoval | 236 | 0.79 | male |
| montane | Jemez Mountains | MSB | 62098 | NM | Sandoval | 236 | 0.79 | female |
| montane | Jemez Mountains | MSB | 62101 | NM | Sandoval | 216 | 0.71 | male |
| montane | Jemez Mountains | MSB | 62102 | NM | Sandoval | 215 | 0.50 | female |
| montane | Jemez Mountains | MSB | 67525 | NM | Sandoval | 193 |  | female |
| montane | Sacramento Mountains | ANSP | 14779 | NM | Otero | 182 |  |  |
| montane | Sacramento Mountains | ANSP | 15771 | NM | Otero | 169 | 0.58 | male |
| montane | Sacramento Mountains | ANSP | 15772 | NM | Otero | 169 | 0.50 | female |
| montane | Sacramento Mountains | ANSP | 15773 | NM | Otero | 178 | 0.47 | female |
| montane | Sacramento Mountains | ANSP | 15774 | NM | Otero | 178 | 0.58 | male |
| montane | Sacramento Mountains | ANSP | 15775 | NM | Otero | 178 | 0.58 | male |
| montane | Sacramento Mountains | ANSP | 15776 | NM | Otero | 182 | 0.61 | male |
| montane | Sacramento Mountains | ANSP | 15777 | NM | Otero | 182 | 0.58 | male |
| montane | Sacramento Mountains | ANSP | 15778 | NM | Otero | 182 | 0.75 | male |
| montane | Sacramento Mountains | ANSP | 15779 | NM | Otero | 214 | 0.58 | male |
| montane | Sacramento Mountains | FT | 358 | NM | Otero | 200 | 0.68 | female |
| montane | Sacramento Mountains | FT | 359 | NM | Otero | 203 | 0.44 | male |
| montane | Sacramento Mountains | MSB | 36142 | NM | Otero | 202 | 0.85 | female |
| montane | Sacramento Mountains | MSB | 37154 | NM | Otero | 202 | 0.35 | male |
| montane | Sacramento Mountains | MSB | 37155 | NM | Otero | 202 | 0.50 | male |
| montane | Sacramento Mountains | MSB | 37323 | NM | Otero | 246 | 0.76 | female |
| montane | Sacramento Mountains | MSB | 37324 | NM | Otero | 246 | 0.84 | female |
| montane | Sacramento Mountains | MSB | 37325 | NM | Otero | 246 | 0.54 | female |
| montane | Sacramento Mountains | MSB | 37326 | NM | Otero | 246 | 0.21 | female |
| montane | Sacramento Mountains | MSB | 41058 | NM | Otero | 230 | 0.50 | female |
| montane | Sacramento Mountains | MSB | 41059 | NM | Otero | 230 | 0.53 | male |
| montane | Sacramento Mountains | MSB | 41060 | NM | Otero | 229 | 0.71 | female |
| montane | Sacramento Mountains | MSB | 41061 | NM | Otero | 229 | 0.71 | female |
| montane | Sacramento Mountains | MSB | 41062 | NM | Otero | 229 | 0.50 | female |
| montane | Sacramento Mountains | MSB | 41063 | NM | Otero | 229 | 0.75 | male |
| montane | Sacramento Mountains | MSB | 41064 | NM | Otero | 229 | 0.50 | female |
| montane | Sacramento Mountains | MSB | 41065 | NM | Otero | 229 | 0.58 | male |
| montane | Sacramento Mountains | MSB | 41066 | NM | Otero | 229 | 0.50 | female |
| montane | Sacramento Mountains | MSB | 61678 | NM | Otero | 194 | 0.50 | male |
| montane | Sacramento Mountains | MSB | 61679 | NM | Otero | 194 | 0.50 | male |
| montane | Sacramento Mountains | MSB | 61680 | NM | Otero | 194 | 0.50 | male |
| montane | Sacramento Mountains | MSB | 61684 | NM | Otero | 196 | 0.50 | female |
| montane | Sacramento Mountains | MSB | 61686 | NM | Otero | 197 | 0.50 | male |
| montane | Sacramento Mountains | MSB | 61687 | NM | Otero | 197 | 0.35 | female |
| montane | Sacramento Mountains | MSB | 61688 | NM | Otero | 198 |  | male |
| montane | Sacramento Mountains | MSB | 61689 | NM | Otero | 198 |  | female |
| montane | Sacramento Mountains | MSB | 61690 | NM | Otero | 198 | 0.35 | male |
| montane | Sacramento Mountains | MSB | 61691 | NM | Otero | 199 | 0.57 | female |
| montane | Sacramento Mountains | MSB | 61692 | NM | Otero | 199 | 0.50 | male |
| montane | Sacramento Mountains | MSB | 61693 | NM | Otero | 200 | 0.42 | male |
| montane | Sacramento Mountains | MSB | 61696 | NM | Otero | 201 | 0.35 | male |
| montane | Sacramento Mountains | MSB | 61700 | NM | Otero | 203 | 0.35 | female |
| montane | Sacramento Mountains | MSB | 61701 | NM | Otero | 203 | 0.46 | female |
| montane | Sacramento Mountains | MSB | 61702 | NM | Otero | 203 | 0.35 | female |
| montane | Sacramento Mountains | MSB | 61703 | NM | Otero | 203 | 0.53 |  |
| montane | Sacramento Mountains | MSB | 61704 | NM | Otero | 203 | 0.47 |  |
| montane | Sacramento Mountains | MSB | 61712 | NM | Otero | 212 | 0.35 | female |
| montane | Sacramento Mountains | MSB | 62095 | NM | Otero | 199 |  | female |
| montane | Sacramento Mountains | USNM | 118798 | NM | Otero | 253 | 0.25 | female |
| montane | Sacramento Mountains | USNM | 119032 | NM | Otero | 250 | 0.61 | male |
| montane | Sacramento Mountains | USNM | 119033 | NM | Otero | 250 |  | female |
| montane | Sacramento Mountains | WNMU | 5695 | NM | Otero | 177 |  |  |
| montane | Sangre de Cristo Mountains | DMNH | 8630 | CO | Las Animas | 162 |  | male |
| montane | Sangre de Cristo Mountains | DMNH | 8631 | CO | Las Animas | 197 |  | female |
| montane | Sangre de Cristo Mountains | DMNH | 8632 | CO | Las Animas | 221 |  | male |
| montane | Sangre de Cristo Mountains | DMNH | 8633 | CO | Las Animas | 221 |  | female |
| montane | Sangre de Cristo Mountains | DMNH | 8634 | CO | Las Animas | 222 |  | male |
| montane | Sangre de Cristo Mountains | DMNH | 8635 | CO | Las Animas | 222 |  | female |
| montane | Sangre de Cristo Mountains | DMNH | 8636 | CO | Las Animas | 222 |  | male |
| montane | Sangre de Cristo Mountains | DMNH | 8637 | CO | Las Animas | 222 |  | female |
| montane | Sangre de Cristo Mountains | DMNH | 8638 | CO | Las Animas | 225 |  | female |
| montane | Sangre de Cristo Mountains | DMNH | 8639 | CO | Las Animas | 225 |  | female |
| montane | Sangre de Cristo Mountains | DMNH | 8640 | CO | Las Animas | 225 |  | female |
| montane | Sangre de Cristo Mountains | DMNH | 8641 | CO | Las Animas | 225 |  | male |
| montane | Sangre de Cristo Mountains | DMNH | 8642 | CO | Las Animas | 225 |  | male |
| montane | Sangre de Cristo Mountains | DMNH | 9065 | CO | Las Animas | 237 |  | male |
| montane | Sangre de Cristo Mountains | FT | 502 | NM | Colfax | 193 | 0.39 | female |
| montane | Sangre de Cristo Mountains | FT | 506 | NM | Colfax | 193 | 0.42 | female |
| montane | Sangre de Cristo Mountains | FT | 507 | NM | Colfax | 193 | 0.50 | female |
| montane | Sangre de Cristo Mountains | FT | 520 | NM | Colfax | 193 | 0.86 | female |
| montane | Sangre de Cristo Mountains | FT | 521 | NM | Colfax | 193 | 0.35 | male |
| montane | Sangre de Cristo Mountains | FT | 528 | NM | Colfax | 194 | 0.35 | male |
| montane | Sangre de Cristo Mountains | FT | 529 | NM | Colfax | 194 | 0.42 | female |
| montane | Sangre de Cristo Mountains | FT | 541 | NM | Colfax | 195 | 0.35 | male |
| montane | Sangre de Cristo Mountains | FT | 542 | NM | Colfax | 195 | 0.79 | male |
| montane | Sangre de Cristo Mountains | FT | 543 | NM | Colfax | 195 | 0.50 | male |
| montane | Sangre de Cristo Mountains | FT | 604 | NM | Mora | 208 | 0.50 | female |
| montane | Sangre de Cristo Mountains | FT | 605 | NM | Mora | 208 | 0.79 | female |
| montane | Sangre de Cristo Mountains | MSB | 4943 | NM | Taos | 175 | 0.60 | male |
| montane | Sangre de Cristo Mountains | TTU | 2388 | NM | Taos | 200 | 0.47 | male |
| montane | White Mountains | ASU | 3132 | AZ | Apache | 182 |  | female |
| montane | White Mountains | ASU | 3133 | AZ | Apache | 184 |  | female |
| montane | White Mountains | ASU | 3136 | AZ | Apache | 186 |  | female |
| montane | White Mountains | ASU | 3137 | AZ | Apache | 182 |  | female |
| montane | White Mountains | ASU | 3650 | AZ | Apache | 186 |  | male |
| montane | White Mountains | ASU | 3651 | AZ | Apache | 188 |  | male |
| montane | White Mountains | FT | 1083 | AZ | Apache | 203 |  | female |
| montane | White Mountains | FT | 1083 | AZ | Apache | 204 |  |  |
| montane | White Mountains | FT | 1084 | AZ | Apache | 203 |  | female |
| montane | White Mountains | FT | 1085 | AZ | Apache | 203 |  | female |
| montane | White Mountains | FT | 1086 | AZ | Apache | 203 |  | male |
| montane | White Mountains | FT | 1087 | AZ | Apache | 203 |  | male |
| montane | White Mountains | FT | 1088 | AZ | Apache | 203 |  | male |
| montane | White Mountains | FT | 1104 | AZ | Apache | 204 |  | female |
| montane | White Mountains | FT | 1105 | AZ | Apache | 204 |  | female |
| montane | White Mountains | FT | 1106 | AZ | Apache | 204 |  | female |
| montane | White Mountains | FT | 1107 | AZ | Apache | 204 |  | female |
| montane | White Mountains | FT | 1134 | AZ | Apache | 205 |  | male |
| montane | White Mountains | FT | 1135 | AZ | Apache | 207 |  | male |
| montane | White Mountains | FT | 1136 | AZ | Apache | 207 |  | female |
| montane | White Mountains | FT | 1136 | AZ | Apache | 208 |  |  |
| montane | White Mountains | FT | 1137 | AZ | Apache | 207 |  | female |
| montane | White Mountains | FT | 1150 | AZ | Apache | 208 |  | male |
| montane | White Mountains | FT | 1151 | AZ | Apache | 208 |  | male |
| montane | White Mountains | FT | 1152 | AZ | Apache | 208 |  | male |
| montane | White Mountains | FT | 1180 | AZ | Apache | 211 |  | female |
| montane | White Mountains | FT | 1181 | AZ | Apache | 211 |  | male |
| montane | White Mountains | FT | 1182 | AZ | Apache | 211 |  | female |
| montane | White Mountains | FT | 1183 | AZ | Apache | 211 |  | female |
| montane | White Mountains | FT | 1184 | AZ | Apache | 211 |  | male |
| montane | White Mountains | FT | 1185 | AZ | Apache | 211 |  | male |
| montane | White Mountains | FT | 1186 | AZ | Apache | 211 |  | female |
| montane | White Mountains | FT | 1214 | AZ | Greenlee | 228 |  | female |
| montane | White Mountains | FT | 1215 | AZ | Greenlee | 228 |  | male |
| montane | White Mountains | FT | 1216 | AZ | Greenlee | 228 |  | female |
| montane | White Mountains | FT | 1224 | AZ | Apache | 230 |  | female |
| montane | White Mountains | FT | 1225 | AZ | Apache | 230 |  | female |
| montane | White Mountains | FT | 1226 | AZ | Apache | 230 |  | male |
| montane | White Mountains | FT | 1235 | AZ | Apache | 255 |  | female |
| montane | White Mountains | FT | 1236 | AZ | Apache | 255 |  | female |
| montane | White Mountains | FT | 1237 | AZ | Apache | 255 |  | female |
| montane | White Mountains | FT | 1238 | AZ | Apache | 255 |  | female |
| montane | White Mountains | FT | 1239 | AZ | Apache | 255 |  | male |
| montane | White Mountains | FT | 1399 | AZ | Apache | 189 |  | male |
| montane | White Mountains | FT | 1559 | AZ | Greenlee | 227 |  | female |
| montane | White Mountains | FT | observed | AZ | Apache | 254 |  |  |
| montane | White Mountains | MNA | 954 | AZ | Apache | 199 |  | male |
| montane | White Mountains | MNA | 2598 | AZ | Apache | 216 |  | male |
| montane | White Mountains | MNA | 2621 | AZ | Apache | 216 |  | male |
| montane | White Mountains | MNA | 2691 | AZ | Apache | 220 |  | male |
| montane | White Mountains | MNA | 2597 | AZ | Apache | 216 |  | female |
| montane | White Mountains | MNA | 2622 | AZ | Apache | 216 |  | female |
| montane | White Mountains | MNA | 2690 | AZ | Apache | 220 |  | female |
| montane | White Mountains | MSB | 40949 | AZ | Apache | 229 | 0.15 | female |
| montane | White Mountains | MSB | 40950 | AZ | Apache | 229 | 0.15 | male |
| montane | White Mountains | MSB | 40951 | AZ | Apache | 229 | 0.58 | male |
| montane | White Mountains | MSB | 40952 | AZ | Apache | 229 | 0.61 | female |
| montane | White Mountains | MSB | 40953 | AZ | Apache | 229 | 0.50 | female |
| montane | White Mountains | MSB | 40954 | AZ | Apache | 229 | 0.79 | male |
| montane | White Mountains | MSB | 40955 | Az | Apache | 229 | 0.82 | female |
| montane | White Mountains | MSB | 40956 | AZ | Apache | 229 | 0.67 | male |
| montane | White Mountains | MSB | 40994 | AZ | Apache | 230 | 0.57 | male |
| montane | White Mountains | MSB | 40995 | AZ | Apache | 230 | 0.17 | female |
| montane | White Mountains | MSB | 40996 | AZ | Apache | 230 | 0.15 | female |
| montane | White Mountains | MSB | 40997 | AZ | Apache | 230 | 0.15 | male |
| montane | White Mountains | MSB | 40998 | AZ | Apache | 230 | 0.50 | female |
| montane | White Mountains | MSB | 86344 | AZ | Apache | 223 | 0.57 |  |
| montane | White Mountains | MSB | 89194 | AZ | Apache | 224 | 0.50 | female |
| montane | White Mountains | MSB | 91627 | AZ | Apache | 225 | 0.58 | female |
| montane | White Mountains | MSB | 91675 | AZ | Navajo | 223 | 0.44 | female |
| montane | White Mountains | MVZ | 56822 | AZ | Greenlee | 264 | 0.50 | male |
| montane | White Mountains | MVZ | 61313 | AZ | Greenlee | 187 |  | male |
| montane | White Mountains | MVZ | 61314 | AZ | Greenlee | 187 |  | female |
| montane | White Mountains | MVZ | 61315 | AZ | Apache | 194 |  | male |
| montane | White Mountains | MVZ | 61316 | AZ | Apache | 194 |  | male |
| montane | White Mountains | MVZ | 61317 | AZ | Apache | 195 |  | female |
| montane | White Mountains | MVZ | 61318 | AZ | Apache | 195 |  | female |
| montane | White Mountains | MVZ | 61319 | AZ | Apache | 195 |  | male |
| montane | White Mountains | MVZ | 61320 | AZ | Apache | 195 |  | male |
| montane | White Mountains | MVZ | 61321 | AZ | Apache | 195 |  | male |
| montane | White Mountains | MVZ | 61322 | AZ | Apache | 196 |  | male |
| montane | White Mountains | SDNHM | 10613 | AZ | Apache | 175 |  |  |
| montane | White Mountains | SDNHM | 10614 | AZ | Apache | 175 |  |  |
| montane | White Mountains | SDNHM | 10630 | AZ | Apache | 177 |  |  |
| montane | White Mountains | SDNHM | 10631 | AZ | Apache | 177 |  |  |
| montane | White Mountains | SDNHM | 10643 | AZ | Apache | 178 |  |  |
| montane | White Mountains | SDNHM | 10659 | AZ | Apache | 180 |  |  |
| montane | White Mountains | SDNHM | 10660 | AZ | Apache | 180 |  |  |
| montane | White Mountains | SDNHM | 10661 | AZ | Apache | 180 |  |  |
| montane | White Mountains | SDNHM | 10662 | AZ | Apache | 180 |  |  |
| montane | White Mountains | SDNHM | 10667 | AZ | Apache | 181 |  |  |
| montane | White Mountains | SDNHM | 10668 | AZ | Apache | 181 |  |  |
| montane | White Mountains | SDNHM | 10669 | AZ | Apache | 181 |  |  |
| montane | White Mountains | SDNHM | 10670 | AZ | Apache | 181 |  |  |
| montane | White Mountains | SDNHM | 10671 | AZ | Apache | 181 |  |  |
| montane | White Mountains | SDNHM | 10681 | AZ | Apache | 182 |  |  |
| montane | White Mountains | SDNHM | 10682 | AZ | Apache | 182 |  |  |
| montane | White Mountains | SDNHM | 10683 | AZ | Apache | 182 |  |  |
| montane | White Mountains | SDNHM | 10690 | AZ | Apache | 183 |  |  |
| montane | White Mountains | SDNHM | 10699 | AZ | Apache | 184 |  |  |
| montane | White Mountains | SDNHM | 10705 | AZ | Apache | 185 |  |  |
| montane | White Mountains | SDNHM | 10707 | AZ | Apache | 187 |  |  |
| montane | White Mountains | SDNHM | 10708 | AZ | Apache | 187 |  |  |
| montane | White Mountains | SDNHM | 10719 | AZ | Apache | 189 |  |  |
| montane | White Mountains | SDNHM | 10723 | AZ | Apache | 191 |  |  |
| montane | White Mountains | SDNHM | 22745 | AZ | Apache | 273 |  |  |
| montane | White Mountains | UA | 897 | AZ | Apache | 173 |  | male |
| montane | White Mountains | UA | 12332 | AZ | Apache | 277 |  |  |
| montane | White Mountains | UA | 15431 | AZ | Apache | 195 |  | female |
| montane | White Mountains | UA | 16828 | AZ | Apache | 273 |  | female |
| montane | White Mountains | UA | 22194 | AZ | Apache | 226 |  | female |
| montane | White Mountains | UA | 24378 | AZ | Apache | 247 |  | female |
| montane | White Mountains | UA | 24379 | AZ | Apache | 244 |  | female |
| montane | White Mountains | UA | 24380 | AZ | Apache | 246 |  |  |
| montane | White Mountains | UA | 26124 | AZ | Apache | 171 |  | male |
| montane | White Mountains | UIMNH (in MSB) | 28305 | AZ | Apache | 185 |  | female |
| montane | White Mountains | UIMNH (in MSB) | 28405 | AZ | Apache | 185 | 0.35 | female |
| montane | White Mountains | UIMNH (in MSB) | 28406 | AZ | Apache | 183 |  | male |
| montane | White Mountains | UIMNH (in MSB) | 29077 | AZ | Apache | 247 | 0.72 | female |
| montane | White Mountains | UIMNH (in MSB) | 29078 | AZ | Apache | 248 | 0.17 | female |
| montane | White Mountains | UIMNH (in MSB) | 50974 | AZ | Apache | 181 | 0.94 | female |
| montane | White Mountains | USNM | 205366 | AZ | Apache | 262 | 0.50 | female |
| montane | White Mountains | USNM | 205373 | AZ | Apache | 263 | 0.50 |  |
| montane | White Mountains | USNM | 205585 | AZ | Apache | 263 | 0.44 | male |
| montane | White Mountains | USNM | 208660 | AZ | Apache | 214 | 0.71 | male |
| montane | White Mountains | USNM | 209296 | AZ | Apache | 213 |  | male |
| montane | White Mountains | USNM | 209297 | AZ | Apache | 213 |  | female |
| montane | White Mountains | USNM | 209298 | AZ | Apache | 213 |  | female |
| montane | White Mountains | USNM | 265988 | AZ | Apache | 292 |  | male |
| montane | White Mountains | USNM | 265989 | AZ | Apache | 299 |  | female |
| valley | Florida River | KU | 16038 | CO | LaPlata | 247 | 0.82 | female |
| valley | Florida River | MSB | 154917 | CO | La Plata | 186 |  | female |
| valley | Florida River | MSB | 155117 | CO | La Plata | 188 |  | female |
| valley | Mora River | NMMNHS | 1228 | NM | Mora | 234 | 0.94 | female |
| valley | Mora River | NMMNHS | 1229 | NM | Mora | 234 | 0.79 | male |
| valley | Rio Chama | KU | 5832 | NM | Rio Arriba | 187 | 0.51 | female |
| valley | Rio Chama | KU | 5833 | NM | Rio Arriba | 195 | 0.35 | male |
| valley | Rio Chama | KU | 5834 | NM | Rio Arriba | 199 | 0.74 | female |
| valley | Rio Chama | KU | 5835 | NM | Rio Arriba | 184 | 0.60 | female |
| valley | Rio Chama | MSB | 58369 | NM | Rio Arriba | 258 | 0.35 | male |
| valley | Rio Grande | MSB | 36119 | NM | Socorro | 240 | 0.35 | female |
| valley | Rio Grande | MSB | 36143 | NM | Socorro | 203 | 0.50 | female |
| valley | Rio Grande | MSB | 36159 | NM | Socorro | 181 | 0.50 | male |
| valley | Rio Grande | MSB | 36160 | NM | Socorro | 173 | 0.50 | female |
| valley | Rio Grande | MSB | 36161 | NM | Socorro | 173 | 0.60 | female |
| valley | Rio Grande | MSB | 36162 | NM | Socorro | 173 | 0.50 | female |
| valley | Rio Grande | MSB | 36164 | NM | Socorro | 195 | 0.35 | female |
| valley | Rio Grande | MSB | 36165 | NM | Socorro | 195 | 0.35 | male |
| valley | Rio Grande | MSB | 36166 | NM | Socorro | 195 | 0.56 | male |
| valley | Rio Grande | MSB | 36167 | NM | Socorro | 195 | 0.57 | male |
| valley | Rio Grande | MSB | 36169 | NM | Socorro | 229 | 0.18 | female |
| valley | Rio Grande | MSB | 36170 | NM | Socorro | 229 | 0.35 | female |
| valley | Rio Grande | MSB | 36171 | NM | Socorro | 229 | 0.35 | male |
| valley | Rio Grande | MSB | 36172 | NM | Socorro | 229 | 0.47 |  |
| valley | Rio Grande | MSB | 36174 | NM | Socorro | 259 | 0.15 | female |
| valley | Rio Grande | MSB | 36175 | NM | Socorro | 259 | 0.15 | female |
| valley | Rio Grande | MSB | 37754 | NM | Socorro | 232 | 0.35 |  |
| valley | Rio Grande | MSB | 37756 | NM | Socorro | 232 | 0.35 |  |
| valley | Rio Grande | MSB | 37757 | NM | Socorro | 232 | 0.35 |  |
| valley | Rio Grande | MSB | 37758 | NM | Socorro | 232 | 0.35 |  |
| valley | Rio Grande | MSB | 41223 | NM | Socorro | 235 | 0.35 | male |
| valley | Rio Grande | MSB | 41224 | NM | Socorro | 236 | 0.35 | female |
| valley | Rio Grande | MSB | 41225 | NM | Socorro | 244 | 0.35 | male |
| valley | Rio Grande | MSB | 41226 | NM | Socorro | 244 | 0.35 | female |
| valley | Rio Grande | MSB | 41227 | NM | Socorro | 244 | 0.35 | male |
| valley | Rio Grande | MSB | 41228 | NM | Socorro | 245 | 0.35 | female |
| valley | Rio Grande | MSB | 41229 | NM | Socorro | 245 | 0.35 | female |
| valley | Rio Grande | MSB | 41230 | NM | Socorro | 245 | 0.35 |  |
| valley | Rio Grande | MSB | 41231 | NM | Socorro | 245 | 0.50 | female |
| valley | Rio Grande | MSB | 41232 | NM | Socorro | 245 | 0.58 | female |
| valley | Rio Grande | MSB | 41233 | NM | Socorro | 246 |  | female |
| valley | Rio Grande | MSB | 41234 | NM | Socorro | 246 | 0.35 | female |
| valley | Rio Grande | MSB | 41235 | NM | Socorro | 246 | 0.35 | female |
| valley | Rio Grande | MSB | 58368 | NM | Bernalillo | 231 | 0.44 | female |
| valley | Rio Grande | MSB | 58370 | NM | Rio Arriba | 255 | 0.35 | male |
| valley | Rio Grande | MSB | 58371 | NM | Rio Arriba | 258 | 0.39 | male |
| valley | Rio Grande | MSB | 62103 | NM | Valencia | 213 | 0.90 | male |
| valley | Rio Grande | NMSU | 13825 | NM | Bernalillo | 240 | 0.21 | female |
| valley | Rio Grande | NMSU | 13826 | NM | Bernalillo | 168 | 0.58 | female |
| valley | Rio Grande | NMSU | 13827 | NM | Bernalillo | 144 | 0.54 | male |
| valley | Rio Grande | NMSU | 13828 | NM | Bernalillo | 144 | 0.50 | male |
| valley | Rio Grande | NMSU | 13829 | NM | Bernalillo | 164 | 0.54 | female |
| valley | Rio Grande | NMSU | 13830 | NM | Bernalillo | 144 | 0.65 | male |
| valley | Rio Grande | USNM | 133601 | NM | Rio Arriba | 175 |  | female |
| valley | Rio Grande | USNM | 133602 | NM | Rio Arriba | 175 | 0.50 | female |
| valley | Rio Grande | USNM | 133603 | NM | Rio Arriba | 176 |  | female |
| valley | Rio Grande | USNM | 133604 | NM | Rio Arriba | 178 |  | male |
| valley | Rio Grande | USNM | 160731 | NM | Socorro | 235 |  | female |
| valley | Rio Grande | USNM | 226911 | NM | Bernalillo | 187 |  |  |
| valley | Sambrito Creek | MSB | 10238 | CO | Archuleta | 206 | 0.18 | male |
| valley | Sambrito Creek | UIMNH (in MSB) | 16896 | CO | Archuleta | 198 | 0.94 | male |
| valley | Sambrito Creek | UIMNH (in MSB) | 16910 | CO | Archuleta | 200 | 0.69 | male |
| valley | Sambrito Creek | UIMNH (in MSB) | 16911 | CO | Archuleta | 201 | 0.94 | male |
| valley | Sambrito Creek | UIMNH (in MSB) | 16912 | CO | Archuleta | 201 | 0.65 | female |
| valley | Sambrito Creek | UIMNH (in MSB) | 16976 | CO | Archuleta | 201 | 0.79 | male |
| valley | Sambrito Creek | UIMNH (in MSB) | 16977 | CO | Archuleta | 201 | 0.94 | female |
| valley | Sambrito Creek | UIMNH (in MSB) | 16978 | CO | Archuleta | 198 |  | male |
