# Supplementary material for: Variation in phenology of hibernation and reproduction in the endangered New Mexico meadow jumping mouse (Zapus hudsonius luteus)
Source: PeerJ. 2015 Aug 6;3:e1138. doi: 10.7717/peerj.1138 (PMC4540022; doi:10.7717/peerj.1138)
Supplement: Table S2 — Reproductive data for female meadow jumping mice (Zapus hudsonius luteus) that were field-evaluated to be pregnant at Bosque del Apache National Wildlife Refuge, Socorro County, New Mexico, 2009–2010 (Wright, 2012; Frey & Wright, 2012). [file peerj-03-1138-s002.docx]

|  |  |  |  | Telemetry data | | | | |  |
| --- | --- | --- | --- | --- | --- | --- | --- | --- | --- |
| Animal number | Date captured | Capture weight (g) | Enlarged mammae | Date became inactive in underground burrow | Days from capture to inactivity | Days of inactivity | Date telemetry ended | Total days from capture to end of telemetry | Notes |
| Z3 | 26-May | 19.0 | not noted | did not | did not | 0 | 14-Jun | 19 | pregnancy unlikely |
| Z6 | 28-May | 20.0 | not noted |  |  |  |  |  | pregnancy unlikely |
| Z10 | 23-Jun | 23.0 | not noted | did not^a^ | did not | 0 | 5-Jul | 12 | pregnancy likely |
| Z20^b^ | 20-Jul | 19.5 | yes | 30-Jul | 10 | 13 | 12-Aug | 23 | pregnancy likely |
| Z22^c^ | 20-Jul | 23.5 | yes | did not | did not | 0 | 4-Aug | 15 | pregnancy likely; predated by owl |
| Z26 | 25 Jul; 26 Jul | 25.0; 26.0 | not noted |  |  |  |  |  | pregnancy likely |
| Z27 | 26-Jul | 31.0 | yes | 30-Jul | 4 | 13 | 12-Aug | 17 | pregnancy likely |
| Z28 | 26-Jul | 28.5 | not noted | 28-Jul | 2 | 15 | 12-Aug | 17 | pregnancy likely |
| Z29 | 27-Jul | 24.0 | yes | 30-Jul | 3 | 26 | 25-Aug | 29 | pregnancy likely; left nest to go to water on last day |
| ^a^The nominal battery life of the transmitters was 21 days, but the signal from this jumping mouse was lost after only 12 days. It is possible the signal was lost because the jumping mouse went to an underground maternal burrow. | | | | | | | | | |
| ^b^Z20 was first caught on 8 July and evaluated as pregnant (19 g) with enlarged mammae, but was not fitted with a radio-collar at that time. | | | | | | | | | |
| ^c^Z22 was first caught on 8 July and evaluated as pregnant (22 g) with enlarged mammae, but was not fitted with a radio-collar at that time. | | | | | | | | | |
